# Optimisation of purification techniques for the preparation of large-volume aqueous solar nanoparticle inks for organic photovoltaics

**DOI:** 10.3762/bjnano.9.60

**Published:** 2018-02-20

**Authors:** Furqan Almyahi, Thomas R Andersen, Nathan A Cooling, Natalie P Holmes, Matthew J Griffith, Krishna Feron, Xiaojing Zhou, Warwick J Belcher, Paul C Dastoor

**Affiliations:** 1 Department of Physics, College of Science, University of Basrah, Iraq; 2 Centre for Organic Electronics, University of Newcastle, University Drive, Callaghan, NSW 2308, Australia; 3 CSIRO Energy Technology, Newcastle, NSW 2300, Australia

**Keywords:** aqueous nanoparticle, crossflow ultrafiltration, miniemulsion, organic photovoltaic, SDS surfactant, surface tension

## Abstract

In this study we have optimised the preparation conditions for large-volume nanoparticle inks, based on poly(3-hexylthiophene) (P3HT):indene-C_60_ multiadducts (ICxA), through two purification processes: centrifugal and crossflow ultrafiltration. The impact of purification is twofold: firstly, removal of excess sodium dodecyl sulfate (SDS) surfactant from the ink and, secondly, concentration of the photoactive components in the ink. The removal of SDS was studied in detail both by a UV–vis spectroscopy-based method and by surface tension measurements of the nanoparticle ink filtrate; revealing that centrifugal ultrafiltration removed SDS at a higher rate than crossflow ultrafiltration even though a similar filter was applied in both cases (10,000 Da *M*_w_ cut-off). The influence of SDS concentration on the aqueous solar nanoparticle (ASNP) inks was investigated by monitoring the surface morphology/topography of the ASNP films using atomic force microscopy (AFM) and scanning electron microscopy (SEM) and photovoltaic device performance as a function of ultrafiltration (decreasing SDS content). The surface morphology/topography showed, as expected, a decreased number of SDS crystallites on the surface of the ASNP film with increased ultrafiltration steps. The device performance revealed distinct peaks in efficiency with ultrafiltration: centrifuge purified inks reached a maximum efficiency at a dilution factor of 7.8 × 10^4^, while crossflow purified inks did not reach a maximum efficiency until a dilution factor of 6.1 × 10^9^. This difference was ascribed to the different wetting properties of the prepared inks and was further corroborated by surface tension measurements of the ASNP inks which revealed that the peak efficiencies for both methods occurred for similar surface tension values of 48.1 and 48.8 mN m^−1^. This work demonstrates that addressing the surface tension of large-volume ASNP inks is key to the reproducible fabrication of nanoparticle photovoltaic devices.

## Introduction

Organic photovoltaics (OPV) are a promising energy technology that utilizes large-scale roll-to-roll (R2R) fabrication techniques (such as slot-die coating, flexographical printing and screen printing) for high-speed production due to the solution-processable nature of the device materials [1–2]. The application of high throughput R2R equipment produces devices which have a short energy payback time and which deliver power at a low levelised cost of electricity [3–4]. In state of the art large scale OPV, the organic materials (polymers, fullerene or other macromolecules) comprising the active layer are dissolved in organic solvents, i.e., chloroform or chlorobenzene [5]. The application of these solvents can be an issue for the large-scale production of OPV due to their toxicity as they are harmful towards both environmental and human health. In reality, water is the eco-friendly solvent and is therefore a more sustainable solvent for upscaling of OPVs [6]. Some low molecular weight alcohols are also frequently used as green solvents in the printing industry, whereas they do have advantages such as ease of process primarily due to much easier removal, they all remain human and environmental exposure limits and are flammable. Currently, it is difficult to synthesise water-soluble semiconducting macromolecules suitable for OPV active layers [7]. Therefore, two methods have been developed for transferring currently utilized semiconducting macromolecules into green solvents: Method 1: The reprecipitation method, herein the semiconductive materials are dissolved in organic solvent which is added to a vigorously stirring alcohol causing the semiconductive materials to precipitation into nanoparticles. OPV prepared from this method have shown great efficiency by reaching 4% [8], however, this method have so far only proven efficient on one donor–acceptor pair. The low versatility of this method as well as with a poor stability of the prepared suspension [9] greatly limits the industrial potential of this method. Method 2: The mini-emulsion method: This method involves ultrasonic mixing of the two-phase organic–aqueous system in the presence of a surfactant, which is commonly sodium dodecyl sulfate (SDS), to produce the mini-emulsion [10]. The presence of free (unbound) SDS in the aqueous solar nanoparticle (ASNP) inks affects several aspects of the fabrication of nanoparticle (NP)-OPV devices; including the packing density of the photoactive particles in the nanoparticle films and thus the formation of cracks or de-wetting areas [11–13]. Previous studies have reported nanoparticle films prepared from inks without purification have exhibited a higher degree of trap states than inks where the excess free SDS has been removed [14]. Residual SDS is generally removed by dialysis which is also applied for concentrating the ASNP inks. A centrifugal ultrafiltration process is a common purification method for preparing small-volume ASNP inks [12,15]. However, this technique is not suitable for preparing the large volumes of inks required to fabricate OPV devices on the roll-to-roll (R2R) scale (for instance, to just fill the reservoir of a typical R2R coating head requires 50 mL of ink with a solids content of 60 mg mL^−1^ before even a centimetre has been coated [16]). Since the supernatant volume loss in any individual centrifuge separation step is large and the removal of SDS requires significant dilution and purification steps, the continual manual intervention required to renew the solvent and continue separation makes this technique impractical for large scale material preparation. In an earlier study, ASNP inks with large volume (100 mL) from 500 mL dispersions were prepared, with the purification of ASNPs from excess-surfactant conducted using a commercial crossflow Millipore filtration system [13,17]. However, the fabrication of NP-OPVs using slot-die coating of these purified inks on a roll-to-roll setup required the addition of an fluorosurfactant (FSO) to improve the wetting of the NP ink onto the substrate [17], indicating that the SDS content had not been optimized in the flow purification study. In principle, it should be possible to control the wetting properties of ASNP inks by tailoring the free-SDS surfactant concentration. However, to date it has been difficult to determine the SDS content of aqueous solar nanoparticle inks, even though it is known that the SDS content has an effect on the performance of resulting NP-OPVs [13,18–19].

In this paper, we investigate how the purification method (centrifuge or crossflow ultrafiltration) influences the wetting of ASNP inks and the performance of the corresponding OPV devices. The purification was monitored by measuring both the concentration of free SDS and the surface tension of the ASNP ink. The effects of these factors are correlated with device efficiencies for both small and large volume ASNP ink purification processes. We demonstrate that it is the surface tension of the resultant ink that is the key factor to determine the efficiency of nanoparticle photovoltaic devices independent of the purification method.

## Results and Discussion

The important parameters when upscaling can be summarized into three areas; size of nanoparticles [20], free SDS concentration [21], and internal morphology of nanoparticles [22] (herein evaluated by solar cell performance).

### Particle size distribution

**Figure 1:**
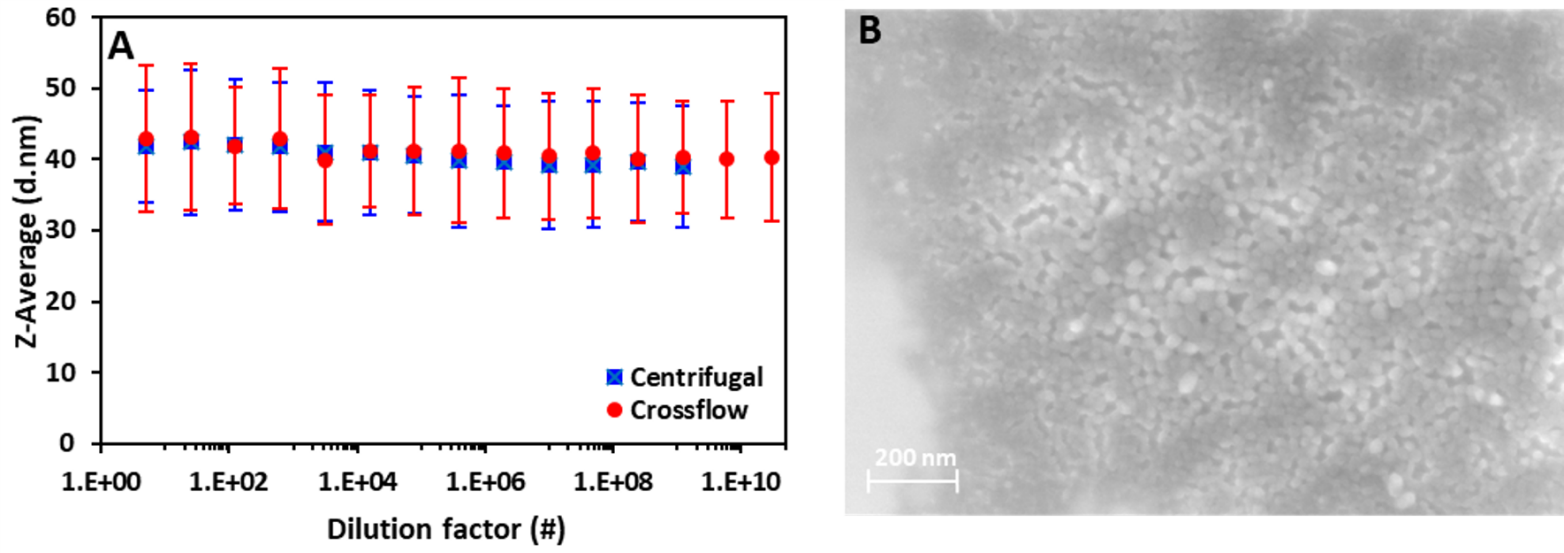
(A) The *Z*-average size of poly(3-hexylthiophene) (P3HT):indene-C_60_ multiadducts (ICxA) NPs dialysed by crossflow and centrifugal ultrafiltration processes vs varying dilution factor. The error bars makes the standard deviation in particle size for the particle population in the sample. (B) SEM image of P3HT:ICxA NPs.

The size of the nanoparticles has an impact on the overall number of layers of particles in an OPV device active layer (as 100 nm thick active layer has been found to be optimal), i.e., smaller particles allow for a more densely packed film, assuming an optimal hexagonal packing of particles [23], which in turn lowers the risk of short circuiting through the active layer. The particle size also influences the overall domain size of donor and acceptor assuming a core–shell structure [15] and therefore greatly influences the efficiency of the prepared devices [24]. Aggregation and sedimentation of ASNP inks as a function of free SDS concentration and applied forces were investigated by measuring the particle size at different dilution factors for inks from both centrifugal and crossflow ultrafiltration methods, as shown in (Figure 1A). There is no evidence of large aggregate formation within the ASNP inks as the particle sizes indicated by the *Z*-average are constant at approximately 41 ± 9 nm as a function of dilution factor (defined in section ‘Preparation of aqueous solar nanoparticle inks’). As such, the particle sizes of the formulated ASNP inks are independent of both dilution factor and ultrafiltration method. The shape of the prepared ASNPs is predominately spherical with a few elongated particles as observed by SEM (Figure 1B).

### Free SDS concentration determination of ASNP inks

**Figure 2:**
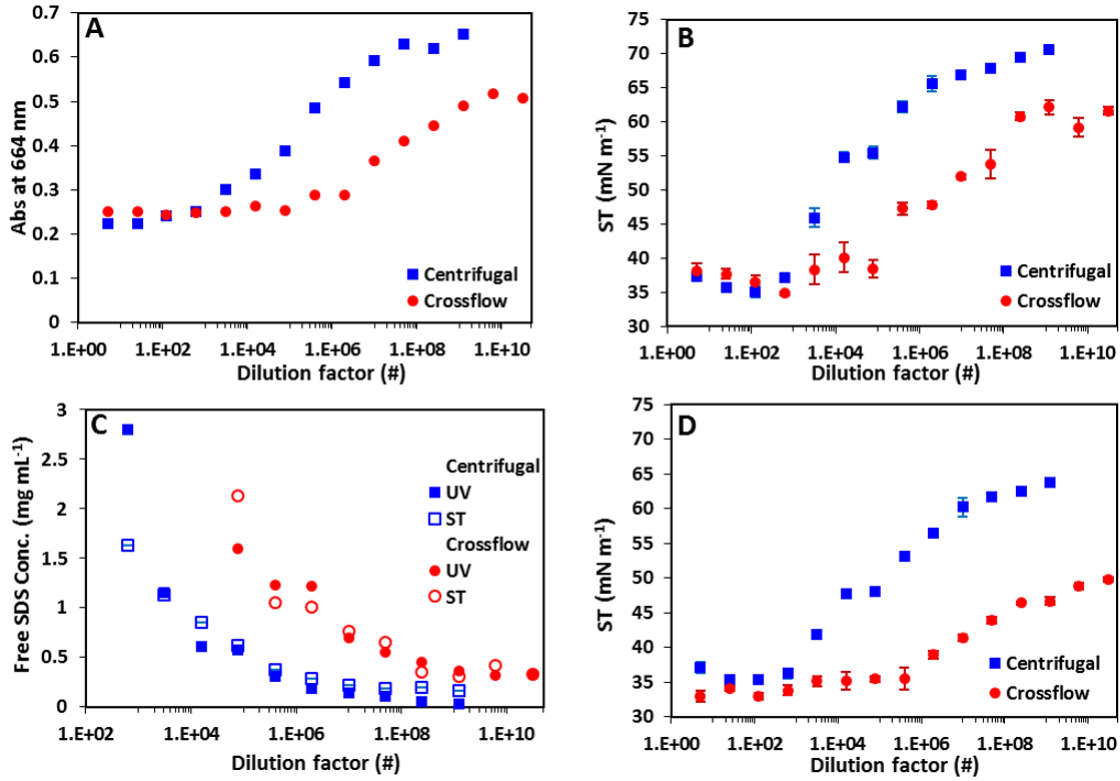
The absorbance at 664 nm of the filtrates of P3HT:ICxA NP inks (A), and surface tension (ST) (B) of the filtrates of P3HT:ICxA NP inks with different dilution factors for centrifugal (blue squares) and crossflow (red circles) ultrafiltration processes. (C) Free SDS concentrations in filtrates vs dilution factor for centrifugal (squares) and crossflow (circles) ultrafiltration processes. (D) The surface tension of NP inks with varying dilution factor for centrifugal (squares) and crossflow (circles) ultrafiltration processes.

The main purpose of the purification of ASNP inks is the removal of excess SDS. For 0.5 mL small-scale nanoparticle inks, the excess SDS is removed by a centrifuge process, which due to volume restrictions is not feasible for large-scale ink preparation where volumes exceed 100 mL. Large-scale ink preparation is therefore conducted by the crossflow ultrafiltration technique with a flow-cartridge. In order to determine the effectiveness of removal of free SDS from the P3HT:ICxA NP inks with increasing dilution factor, the SDS concentration in the filtrates from the purification of P3HT:ICxA NP inks have been determined for both ultrafiltration processes by a UV–vis spectrophotometric method as shown in Figure 2. Figure 2A shows the absorbance values of methylene blue (MB) at 664 nm for the filtrates of ASNP inks that were purified by the centrifugal and the crossflow ultrafiltration techniques with dilution factors of 1–1.2 × 10^9^ and 1–3.1 × 10^10^, respectively. Commonly for both techniques the graphs can be divided into three regions, initially (low dilution factor) the curves appear flat with an absorption of 0.2–0.25 due to an SDS concentration higher than 1 mg mL^−1^, which saturates the coordination complex chemistry and thus provides an upper limit for detection with this technique. This stage is followed by an increase from an absorption of 0.25 to 0.5–0.6; corresponding to an SDS concentration of 0.30–0.35 mg mL^−1^ for centrifuge and crossflow ultrafiltration, respectively (Supporting Information File 1, Figure S1A). In the third region, the curves once again appear flat indicating that we are continuously removing filtrate with a fixed SDS concentration of 0.3–0.35 mg mL^−1^ even when dialysing high dilution factor inks. This result is not a measurement detection limit as observed for the saturation at high SDS content as the calibration samples (Supporting Information File 1, Figure S1B) clearly show detection of SDS at much higher absorbance (lower SDS content) than where the ASNP ink saturate. Thus it is clear the samples reach a fixed SDS content of 0.3–0.35 mg mL^−1^ beyond which no further SDS can be removed with ultrafiltration. Furthermore, as a comparison, surface tension (ST) measurements have been used to determine the free SDS concentration in the filtrates of P3HT:ICxA NP inks with varying dilution factors for each of the purification techniques, as shown in Figure 2B. For the centrifugal and crossflow ultrafiltration, the filtrates with low dilution factors have lower constant surface tensions, which then increase sharply with increasing dilution factors before plateauing at high dilution factor. Using the calibration curve for absorption vs SDS concentration (Supporting Information File 1, Figure S1A) and surface tension vs free SDS concentration curve (Supporting Information File 1, Figure S1B), we created Figure 2C, which show the concentration of SDS in the filtrate as a function of dilution factor for both methods. As seen from Figure 2C the concentration values determined using the UV absorption and surface tension methods are in good agreement. Moreover, both methods clearly show that the purification of ASNP inks using centrifugal ultrafiltration proceeds faster than using crossflow ultrafiltration, indicating a higher physical partition coefficient for separation at the filtration membrane using the centrifuge process.

Figure S2 and Figure S3 (see Supporting Information File 1) show photographs of films coated from ASNP ink with the various dilution factors. It is clear that ASNP inks with low dilution factor (1–625) and (1–3.9 × 10^5^) exhibit poor wetting properties on pre-cleaned glass slides. Moving towards higher dilution factors leads to an improvement in the wetting properties of ASNP inks, however, with excessive SDS removal the wetting properties of the ASNP inks again decreases. Further investigation (Figure 2D) shows that the surface tension of ASNP inks with lower dilution factors than 625 were roughly constant (33.6–36 mN m^−1^) for both purifications. With higher dilution factors, we observe an increase in surface tension, indicating that the concentration of free SDS is reduced. The maximal surface tension of ASNP inks reached was 64 and 50 mN m^−1^ for the centrifugal and the crossflow purifications, respectively.

### Influence of free-SDS surfactant on the surface morphology/topography of ASNP films

**Figure 3:**
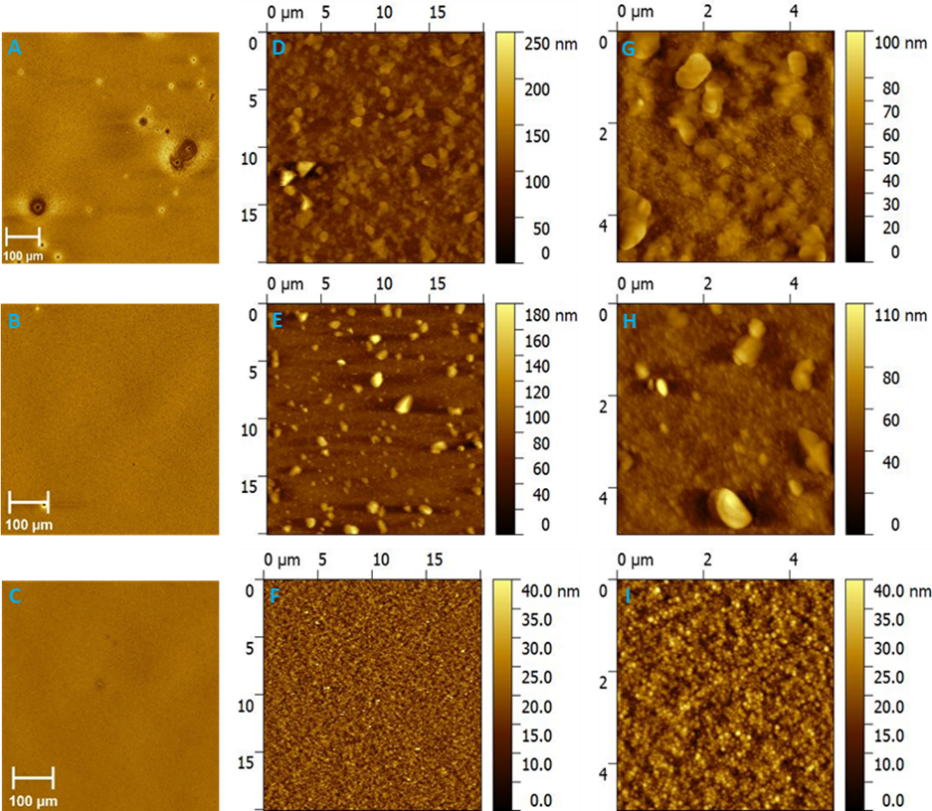
Optical microscopy (A–C), scale bar 100 µm, AFM images with scale bar 20 × 20 µm (D–F) and 5 × 5 µm (G–I) of P3HT:ICxA NP films with dilution factors 625 (A,D,G), 7.8 × 10^4^ (B,E,H) and 9.8 × 10^6^ (C,F,I) for centrifugal ultrafiltration.

**Figure 4:**
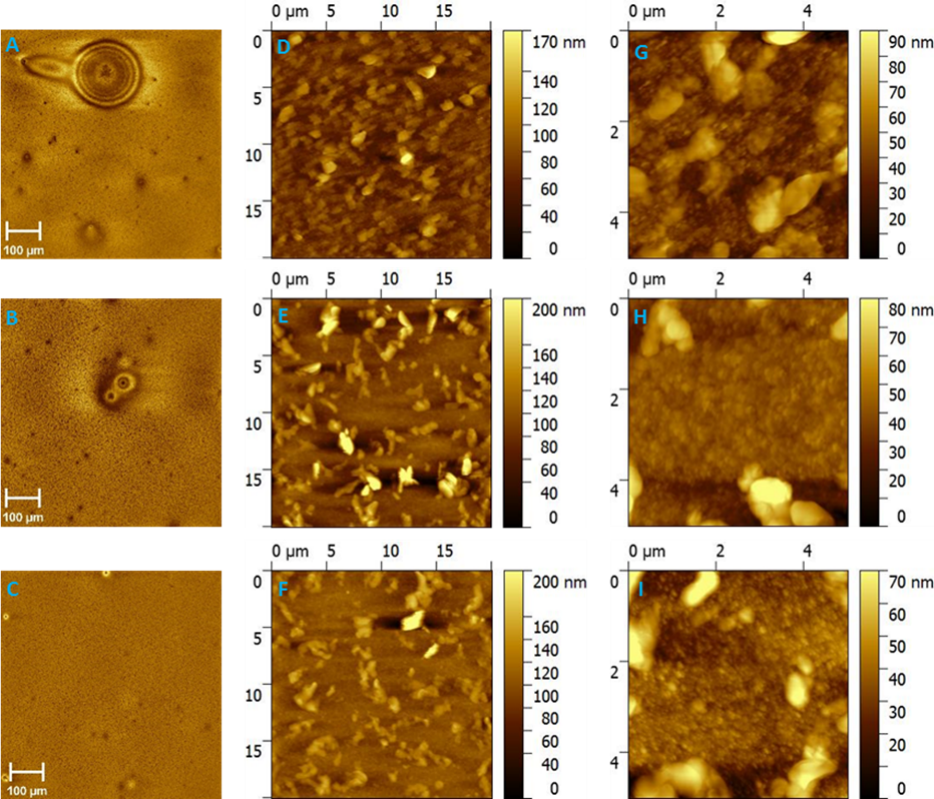
Optical microscopy (A–C), scale bar 100 µm, AFM images with scale bar 20 × 20 µm (D–F) and 5 × 5 µm (G–I) of P3HT:ICxA NPs with dilution factors 1.6 × 10^4^ (A,D,G), 9.8 × 10^6^ (B,E,H) and 6.1 × 10^9^ (C,F,I) for crossflow ultrafiltration.

Optical microscopy and atomic force microscopy (AFM) were conducted for ASNP films with low, medium and high dilution factors; photographs illustrating the film forming ability for all inks can be seen in Supporting Information File 1, Figure S2 and Figure S3. Figure 3A–C shows the optical microscopy images of the as spun ASNP films with dilution factors of 625, 7.8 × 10^4^ and 9.8 × 10^6^ following centrifugal ultrafiltration. The large aggregates present in the film prepared from an ink with a dilution factor of 625 are no longer observed once a highly purified ink with a dilution factor of 9.8 × 10^6^ is utilized and thus we ascribe the observed aggregates to SDS crystallites which are eliminated with SDS removal. When using crossflow ultrafiltration, the ASNP films with dilution factors of 1.6 × 10^4^, 9.8 × 10^6^ and 6.1 × 10^9^ all show aggregates on their surfaces (Figure 4A–C). These aggregates were analysed by energy dispersion X-ray spectroscopy (EDX, Supporting Information File 1, Figure S4). It is evident that the aggregates on the surfaces consist of sulphur, sodium, oxygen and carbon which are the building blocks for SDS, whereas a region with no aggregates show no peak for sodium. Thus, the aggregates are attributed to SDS, since this is the only chemical species present in the ink that contains sodium and oxygen. To further investigate the insights into the nanoscale surface morphologies, AFM was conducted on the same ASNP films, the investigations were conducted on homogenous regions of the NP films rather than on regions dominated by SDS aggregates. The AFM images of P3HT:ICxA NP films are presented in Figure 3D–F with scale 20 × 20 µm and Figure 3G–I with scale 5 × 5 µm for ASNP with centrifugal ultrafiltration. The ASNP films with dilution factor of 625 and 7.8 × 10^4^ revealed multiple SDS aggregates and nanoparticle regions whereas increasing the dilution factor to 9.8 × 10^6^ leads to a NP surfaces without SDS aggregates. With the crossflow purification, the AFM images of ASNP films with dilution factors of 1.6 × 10^4^, 9.8 × 10^6^ and 6.1 × 10^9^ are shown in Figure 4D–F and Figure 4G–I with a scale of 20 × 20 µm and 5 × 5 µm, respectively. In contrast with centrifugal filtration, it is difficult to distinguish the NP regions in the AFM images of the crossflow filtered films prepared with dilution factors of 1.6 × 10^4^; suggesting that the high SDS content of these films is obscuring almost all of the film surface. However, with higher dilution factors of up to 6.1 × 10^9^, the NP regions in the film are more clearly distinguishable and dominate the surface. Thus, although increasing the dilution factor leads to a reduction of the surface SDS concentration for both ultrafiltration processes, centrifugal purification removes SDS much more efficiently than crossflow purification.

### Effect of the ASNP ink purification on NP-OPV performance

NP-OPV devices were successfully fabricated from P3HT:ICxA ASNP inks with dilution factors of 1–1.2 × 10^9^ and 3.9 × 10^5^–3.1 × 10^10^ for centrifugal and crossflow ultrafiltration inks, respectively. The device was prepared in normal geometry in order to determine the optimal SDS concentration based on OPV device performance. Supporting Information File 1, Figure S5A–D shows the *J*–*V* characteristics for dried and annealed NP-OPV devices for the centrifugal and the crossflow ultrafiltration techniques (summarized in Supporting Information File 1, Table S1).

**Figure 5:**
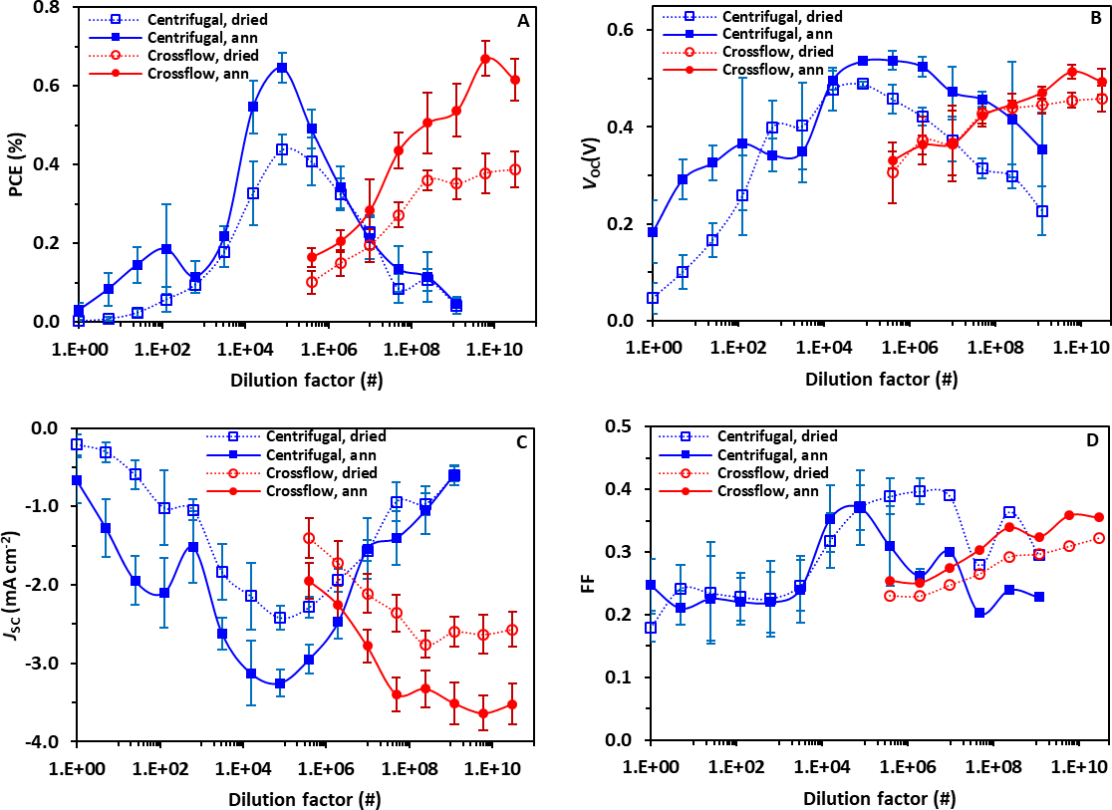
The performance parameters (PCE, *V*_oc_, *J*_sc_ and FF) of P3HT:ICxA NP OPV devices for crossflow and centrifugal ultrafiltration methods plotted as red and blue lines, respectively. Solid line refers to annealed devices (140 °C, 4 min), and dotted line refers to dried (110 °C, 10 min) devices. The error bars represent the standard deviation of an average of 12 devices per sample.

Previous work by Bag et al. has suggested that lower SDS concentration for ASNP inks should lead to more efficient devices due to SDS hindering hole transport [14]. By contrast, in this study, plotting the OPV characteristics (power conversion efficiency (PCE), open-circuit voltage (*V*_OC_), short-circuit current (*J*_SC_) and fill factor (FF)) versus dilution factor (Figure 5A–D) reveals a clear peak in efficiency as a function of dilution factor for inks purified using centrifugal ultrafiltration. A maximum efficiency is observed at a dilution factor 7.8 × 10^4^ with a PCE of 0.65%, while efficiencies for dilution factors of 1.6 × 10^4^ and 3.9 × 10^5^ drop to 0.55% and 0.49%, respectively, and continue to decrease when moving towards both dilution extremes. A similar pattern is obtained for the inks purified by the crossflow ultrafiltration with a low PCE of 0.16% observed for a dilution factor of 3.9 × 10^5^ rising to a peak performance of 0.67% at a dilution factor 6.1 × 10^9^, after which performance decreases slightly to 0.62% at 3.1 × 10^10^. Furthermore, the observation that similar peak efficiencies are obtained at two very different dilution factors is consistent with the earlier observation that SDS is removed at different rates using the two ultrafiltration processes, but may indicate that both techniques produce inks with similar optimized properties at different dilution factors.

**Figure 6:**
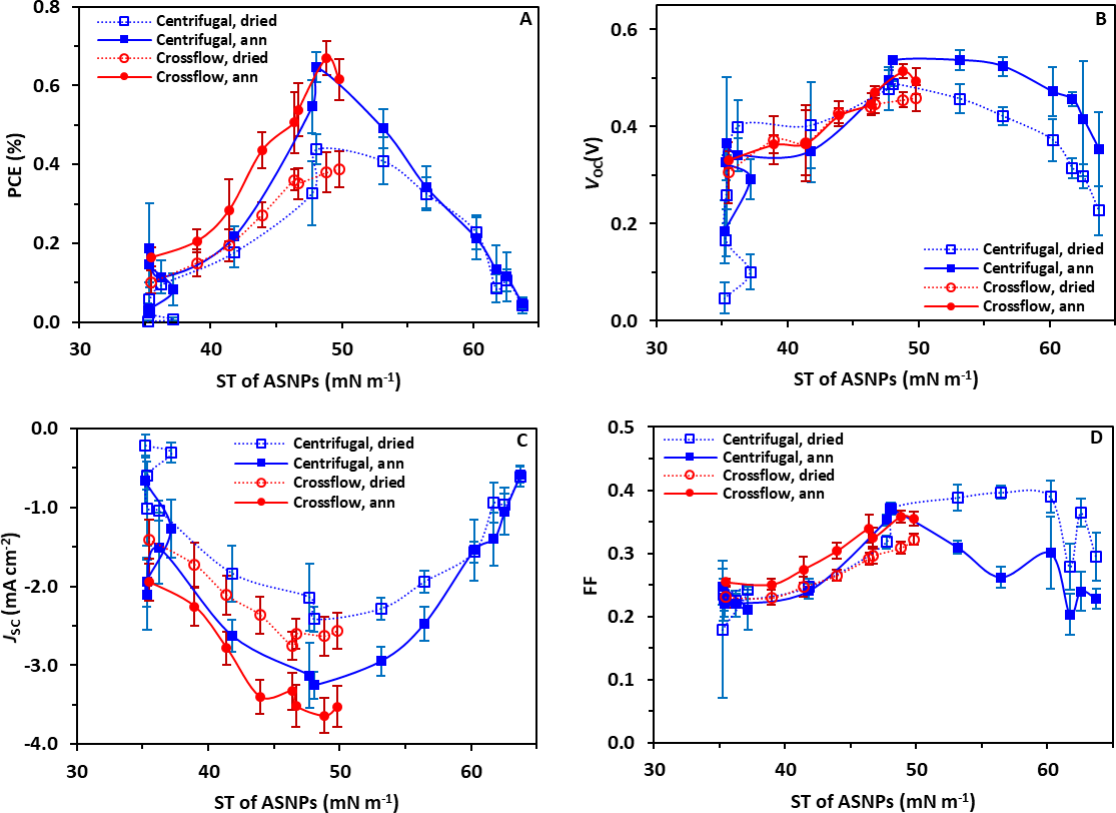
OPV performance showing (A) PCE, (B) *V*_oc_, (C) *J*_sc_ and (D) FF values of NP-OPVs versus surface tension (ST) of P3HT:ICxA NPs dialysed by crossflow and centrifugal process (A–D), respectively. Solid line refers to annealed devices, and dot line refer to dried devices.

To evaluate whether this effect is due to the ink or the purification technique, the efficiency parameters (PCE, *V*_OC_, *J*_SC_ and FF) were plotted as a function of surface tension instead of dilution factor (Figure 6A–D). Figure 6A shows the PCEs of NP-OPV devices versus surface tension of ASNP inks for both purification methods and a clear correlation can be observed, with device efficiencies for inks from both purification processes peaking at a surface tension of approximately 48 mN m^−1^. In contrast, low surface tension of ASNP inks with high free SDS concentrations result in less wetting and less uniform films. The increased surface tension of ASNP inks towards 48 mN m^−1^ were caused by decreasing the free-SDS concentration of inks, and the enhanced efficiency of NP-OPV devices can be attributed to improved wetting properties between ASNP inks and the underlying PEDOT:PSS layer. The efficiency drop observed at higher surface tensions are ascribed to a reduction in the ink wetting properties as observed in Supporting Information File 1, Figure S2 and Figure S3.

The observation that there is an optimal surface tension of 48–49 mN m^−1^ for peak device performance regardless of purification technique is important and reveals that controlling the wetting properties is the dominant parameter in determining the performance of NP-OPV devices at scale. The changing wetting properties for each DF required the spin coating procedure for each device type to be optimized in order to achieve similar layer thicknesses of 105 ± 5 nm. The uniformity of active layer thicknesses was investigated by measuring the reflectance of the prepared devices (seen in Supporting Information File 1, Figure S6). The reflectance measurements illustrate uniform absorption across devices with very different current densities, meaning that the *J*_SC_ trends are clearly not a function of reduced absorption.

The real challenge in the OPV community is the upscaling of devices from small area lab-scale devices prepared by spin-coating to large area R2R prepared devices. We have chosen to utilize HTL Solar as our PEDOT:PSS for this study as it has a PEDOT to PSS ratio of 1:2.5 which is the same as PH1000 which are being used frequently for R2R prepared large area devices [16,25–27]. Moreover, HTL Solar has a lower conductivity than PH1000 which reduces/eliminates cross-over between cells on the same substrate. Based on the PEDOT/PSS ratio being the same, we expect, the wetting properties to be similar for our NP-inks on both types of PEDOT:PSSes. During upscaling the deposition technique would also be changed from spin-coating to slot-die coating which might also have an impact on film quality.

## Conclusion

Herein we have demonstrated ASNP purification through two different ultrafiltration processes (centrifuge and crossflow-based techniques). It is clearly demonstrated that the rate for free-SDS removal varies between the two techniques even though a similar filter was used (10,000 *M*_w_ cut-off). In particular, inks purified by centrifuge ultrafiltration reached an optimal surface tension of 48 mN m^−1^ at a dilution factor of 7.8 × 10^4^ whereas ink purified by crossflow reached an optimal surface tension of 48.8 mN m^−1^ at a dilution factor of 6.1 × 10^9^. It has been shown that the efficiencies of NP-OPV devices vary with the dilution factor and is governed by the wetting properties of ASNP inks. We find that P3HT:ICxA NP inks having a surface tension of 48 and 48.8 mN m^−1^ produce NP-OPV devices having a masked average PCE of 0.65 and 0.67% (0.70 and 0.73% maximum) for centrifugal and crossflow purification, respectively. Therefore, efficient preparation conditions for either purification process of ASNP inks can be simply monitored through the surface tension of the ASNP inks as the key parameter.

## Experimental

### Materials and equipment

Poly(3-hexylthiophene) (P3HT) (*M*_n_ 20 kDa) and ICxA were synthesised in house according to the literature [28], with this batch of ICxA consisting of a mixture of 36% ICMA, 51% ICBA and 13% ICTA [16,27–28]. Sodium dodecyl sulphate (SDS) with 98% purity was purchased from Sigma-Aldrich. Poly(ethylenedioxythiophene):poly(styrene sulfonate) (PEDOT:PSS) Clevios HTL Solar was purchased from Heraeus. For the ultrafiltration process, centrifugal filter tubes (for small-volume) and Vivaflow 200 crossflow cassette (for large-volume) were supplied by Vivaproducts and Sartorius, respectively, each equipped with a poly(ethersulfone) (PES) membrane with a cut-off at 10 kDa (MWCO). A Hielscher ultrasound type UIP1000hdT (1000 W, 20 kHz) with a 22 mm diameter sonotrode was used to prepare the mini-emulsion for large-volume nanoparticle inks. Layer thicknesses were measured by a KLA–Tencor Alpha step 500 surface profilometer.

### Preparation of aqueous solar nanoparticle inks

In accordance with the modified mini-emulsion process [10–11], the formulation of ASNP inks based on P3HT:ICxA (1:0.8) in large batch (100 mL) comprised several procedural steps. The first step is preparation of the organic phase (≈170 g) by dissolving 6 g of P3HT:ICxA in 112 mL of chloroform (CF) (≈54 mg mL^−1^) at 35 °C and 700 rpm on hotplate, and independently 6.6 g of SDS surfactant is dissolved in 560 mL of Milli-Q water (≈12 mg mL^−1^) to form the aqueous phase (≈566 g). The second step is macro-emulsion formation (≈736 g), which is the vigorous mixing of organic and aqueous solutions with stirring at 1200 rpm and 33 °C for 60 min. The third step is formation of a mini-emulsion (≈736 g) by ultrasonic treatment of the macro-emulsion (10 min, 60% amplitude and 450–500 W). As a fourth step, removal of chloroform was conducted at 65 °C and 1200 rpm for 4 h to produce aqueous nanoparticle P3HT:ICxA dispersions (≈500 g). The final step is the ultrafiltration process to remove excess surfactant using two types of purification methods, and to concentrate to 6 wt % solids content. In this step, the small-volume ultrafiltration (2.5 mL) was conducted by centrifuge (3550 rpm with controlling the time to concentrate ASNP inks to 0.5 mL) called a centrifugal ultrafiltration process, whilst the crossflow ultrafiltration process that is comprised of a Vivaflow 200 system involving pump with 1.25 bar pressure was used for large-volume inks (250 mL). The ultrafiltration process was performed in a series of sequential steps, with each step increasing the dilution of SDS by refreshing the ink solution with pure solvent such that each purification step was commenced with an identical initial dilution and completed with an identical volume to provide a constant dilution factor. The initial volume of ASNP dispersions were 250 mL or 2.5 mL for inks purified by the crossflow and centrifugal ultrafiltration processes, respectively. In any single filtration step, these volumes were then purified through ultrafiltration until final volumes of 50 mL and 0.5 mL were obtained, providing a dilution factor of 5. This process was then continually re-iterated with the addition of fresh solvent to provide a series of ASNP ink samples where the dilution factors (DF) varied exponentially, with the exponent being the number of dilution steps (DF = 5^0^, 5^1^, 5^2^ …, 5^15^), where dilution factor of 5 is the ratio between the initial volume and the concentrated volume. Through this repeated dilution procedure, P3HT:ICxA nanoparticle inks with dilution factor of (1 to 1.2 × 10^9^ (5^13^)) and (1 to 3.1 × 10^10^ (5^15^)) were prepared and purified by centrifugal and crossflow methods, respectively. The removal of SDS and concentration of NP inks therefore occurred in the same equivalent volumes for both purification methods, even though the crossflow technique provided a much larger final sample. For large volume NP ink production, 3 mL of ASNP ink was collected at each dilution step for surface tension measurements, the reduced total ink volume was taken into account when adding water by reducing the volume of added water before the next ultrafiltration step. In addition to the preparation of ASNP inks, filtrates were collected at each dilution step for both purification methods. For small volume, 0.5 mL of ASNP inks were collected at each dilution step while the collected filtrate was 2 mL.

### Nanoparticle characterisation

#### Particle size

Particle sizes were determined by dynamic light scattering (DLS) (Zetasiser Nano-ZS ZEN3500, Malvern Instruments) with a 633 nm laser and a backscatter detector angle of 173°. Samples for DLS were prepared by diluting 5 µL of NP ink in 10 mL Milli-Q water in order to reduce the solids content from 6 to 0.003%.

#### Free SDS concentration

**UV-vis spectroscopy:** An ultraviolet–visible absorption spectrophotometer (UV–vis, Varian Cary 6000i) was used to determine the free SDS concentration. This method works by measuring the absorption of methylene blue (MB) and monitoring its reduced absorption after forming a complex with free SDS [29]. Measurements were conducted on samples consisting of 1 mL of filtrate mixed with 2 mL of a 22.5 µM MB stock solution. The UV–vis spectra of the reference (1 mL of water and 2 mL MB stock solution) and calibration standards (SDS solutions with concentrations 0.01, 0.05, 0.1, 0.5, 1,.., 3.5 mg mL^−1^) are shown in Supporting Information File 1, Figure S1A. The absorbance of MB at 664 nm decreased when increasing the SDS surfactant concentration, as shown in the calibration curve in Supporting Information File 1, Figure S1B. Hence free SDS concentration for unknowns were calculated from this calibration curve.

**Surface tension:** An Attension Theta optical tensiometer (Bionic Scientific Co.) was used to record drop images and automatically analyse the drop shape (pendant drop method using OneAttension software) of ASNP inks and their filtrates in order to measure their surface tension.

**Film inspection:** An Asylum Research Cypher atomic force microscope (AFM) operated in AC mode was used to probe the nanoparticle films. Film samples for AFM were spin-coated on quartz glass substrates at 2000 rpm for 1 min. A Zeiss Sigma ZP field-emission scanning electron microscope (FESEM) was used to image the nanoparticle films (operating at accelerating voltage of 2 kV and magnification ranges of 10,000–100,000×), with concurrent energy dispersive X-ray spectroscopy (EDX) measurements. For SEM, NP films were coated on conductive silicon substrates by spin coating, where 2.5 µL of NP ink was diluted in 12.5 µL of water and spun at 3000 rpm speed with 112 rpm/s acceleration for 1 min.

#### Device fabrication

On pre-cleaned (water, acetone and isopropanol) patterned ITO glass substrates PEDOT:PSS (HTL Solar) films were spin-coated at 5000 rpm for 1 min and baked for 30 min at 140 °C (film thickness of 35 ± 5 nm). On top of the PEDOT:PSS layer, the P3HT:ICxA ASNP inks (dilution factor of 625–1.2 × 10^9^ and 3.9 × 10^5^–3.1 × 10^10^ for centrifugal and crossflow ultrafiltration, respectively) were used to fabricate films with thickness of 105 ± 6 nm. These films were spin-coated and dried at 110 °C for 5 min in air, followed by an additional drying step at 110 °C for 5 min in nitrogen glove box. Under vacuum (2 × 10^−6^ Torr) calcium (film thickness ≈30 nm) and aluminium (film thickness ≈100 nm) electrodes (Ca/Al) were thermally evaporated.

#### Film thickness

The Film thickness of the active layer was determined based on an average of 4 scans by profilometry (KLA-Tencor Alpha-step 500 surface profilometer) over a scratch on annealed (140 °C for 4 min) spin coated samples on a glass substrate. The spin coating recipe was adjusted for each sample to obtain the desired thickness.

Reflectance of the prepared devices was measured on a Varian Cary 6000i with an integrating sphere.

#### Device testing

The NP-OPV devices had a masked area of 3.8 mm^2^ and were tested under a nitrogen atmosphere in glovebox before and after annealing at 140 °C for 4 min. Current density–voltage (*J*–*V*) measurements were performed using Keithley 2400 source meter under illumination of a Newport Class AAA solar simulator with an AM 1.5 spectrum filter. The light intensity was calibrated to 100 mW cm^−2^ using a silicon reference solar cell (FHG-ISE).

## Supporting Information

File 1 Supporting Information.
