# Supplementary material for: Optimisation of purification techniques for the preparation of large-volume aqueous solar nanoparticle inks for organic photovoltaics
Source: Beilstein J Nanotechnol. 2018 Feb 20;9:649–59. doi: 10.3762/bjnano.9.60 (PMC5827634; doi:10.3762/bjnano.9.60)
Supplement: File 1 — Supporting Information. [file Beilstein_J_Nanotechnol-09-649-s001.pdf]

Supporting Information

for

Optimisation of purification techniques for the preparation of large-volume aqueous solar nanoparticle inks for organic photovoltaics

Furqan Almyahi[1,2], Thomas R. Andersen[*2], Nathan A. Cooling[2], Natalie P. Holmes[2], Matthew J. Griffith[2], Krishna Feron[2,3], Xiaojing Zhou[2], Warwick J. Belcher[2] and Paul C. Dastoor[2]

Address: [1]Department of Physics, College of Science, University of Basrah, Iraq, [2]Centre for Organic Electronics, University of Newcastle, University Drive, Callaghan, NSW 2308, Australia and [3]CSIRO Energy Technology, Newcastle, NSW 2300, Australia

Email: Thomas R. Andersen - Thomas.andersen@newcastle.edu.au

*Corresponding author

Supporting Information

The surface tension of water decreases with an increase in SDS concentration until a saturation point is reached, the critical micelle concentration (CMC) is located at ≈2.5 mg/mL as a shown in **Figure S1**.

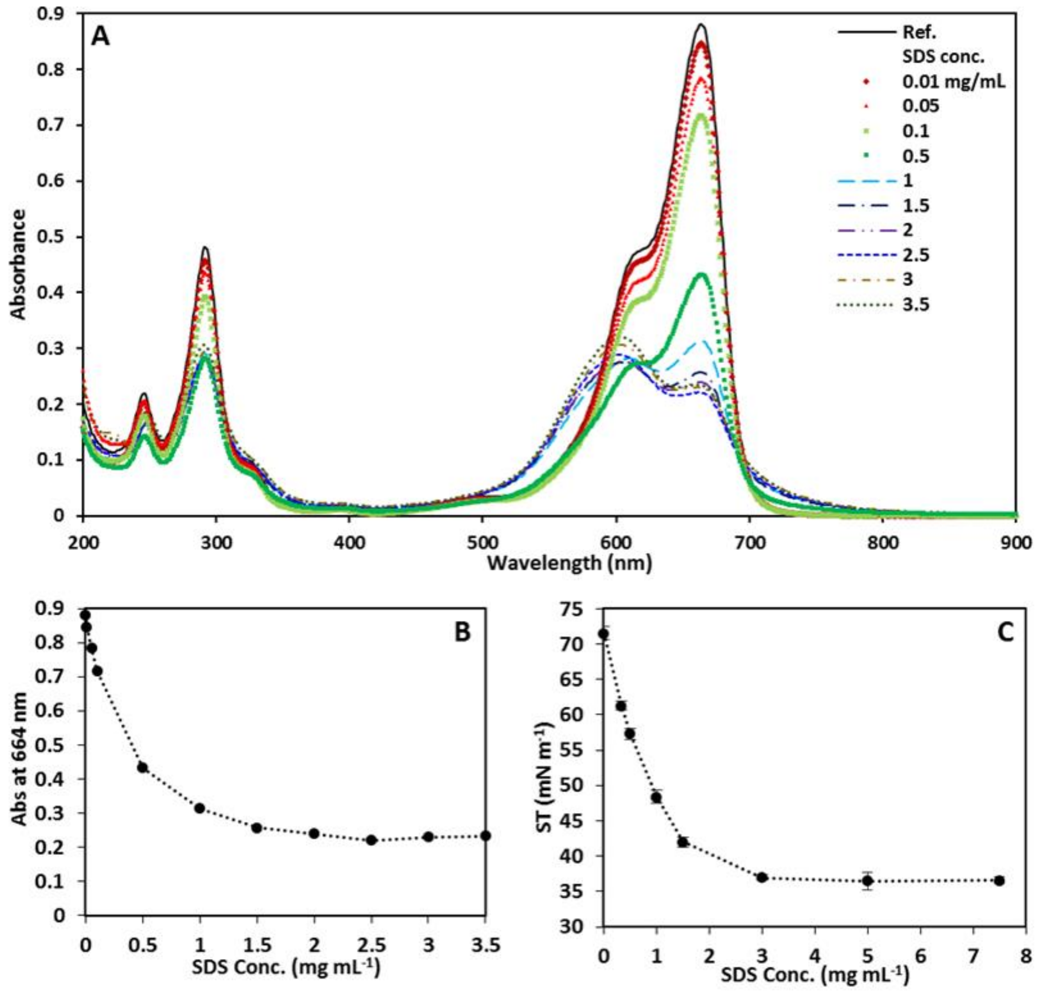

Figure S1: (A) The UV–vis spectra of the reference (the mixture of 2 mL of MB solution (22.5 μM) and 1 mL of water) and calibration standards (SDS solutions with concentrations 0.01, 0.05, 0.1, 0.5, 1,.., 3.5 mg mL^{-1}). Calibration curves: The absorbance at 664 nm (B) and surface tension (C) of SDS solution vs concentration.

Photographs of P3HT:ICxA NP films with dilution factor of 1–1.2 × 10^9 and 1–3.1 × 10^{10} has been shown in Fehler! Verweisquelle konnte nicht gefunden werden. and Fehler! Verweisquelle konnte nicht gefunden werden. for crossflow and centrifugal ultrafiltration processes, respectively. It is clear that the low dilution factor inks form NP films with de-wetting and non-close packing differ from those high dilution factors due to the balanced difference between electrostatic repulsion, attractive van der Waals and capillary forces [1,2]. In contrast, those with higher dilution factor have some cavities due to the change in surface charge density of and between nanoparticles that are varying their surface tensions.

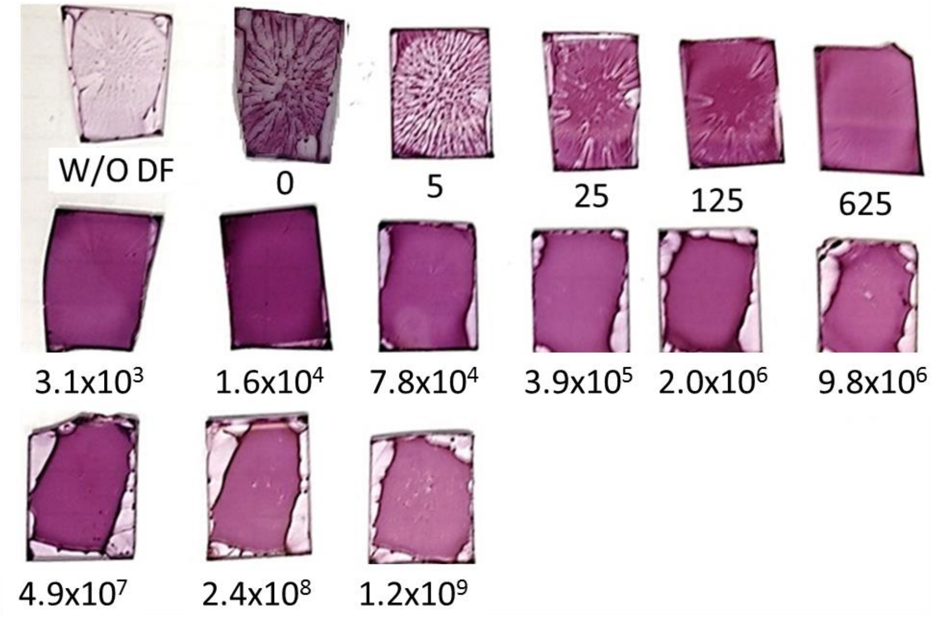

Figure S2: Photograph of P3HT:ICxA NP films with varying dilution factor centrifugal process.

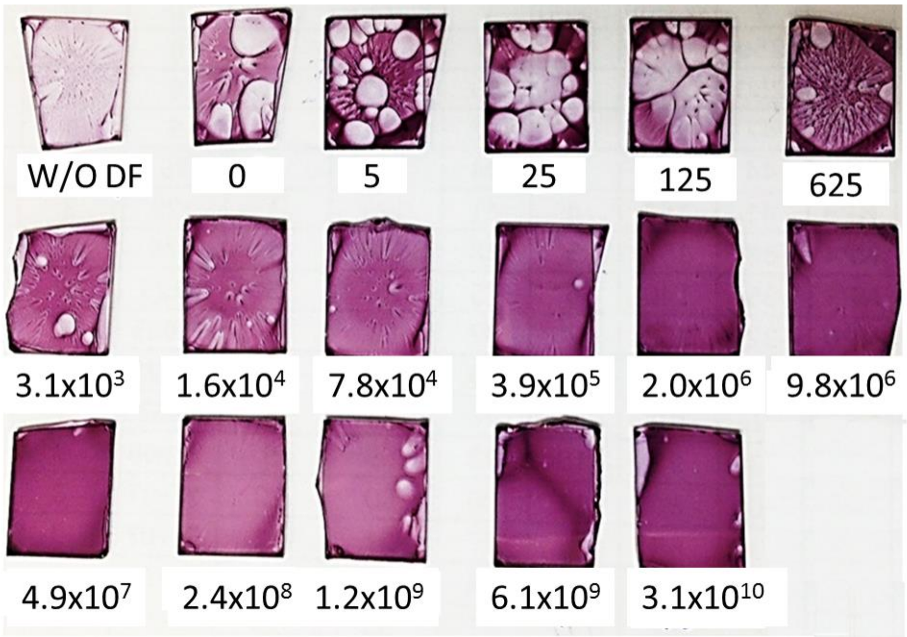

Figure S3: Photograph of P3HT:ICxA NP films with varying dilution factor crossflow process.

SEM images of P3HT:ICxA NP film with dilution factor 25 are presented in Fehler! Verweisquelle konnte nicht gefunden werden., and it can be clearly to distinguish between the NPs and SDS aggregates with increased SEM magnification. The EDX spectral analysis of the SDS crystallite and nanoparticle film region shows sulphur (S), carbon (C), oxygen (O) and sodium (Na) peaks in addition to silicon peak that belong to silicon substrate. The content of S, C, O and Na in the SDS crystallite was higher than of those in NP film region due to their association with the SDS surfactant.

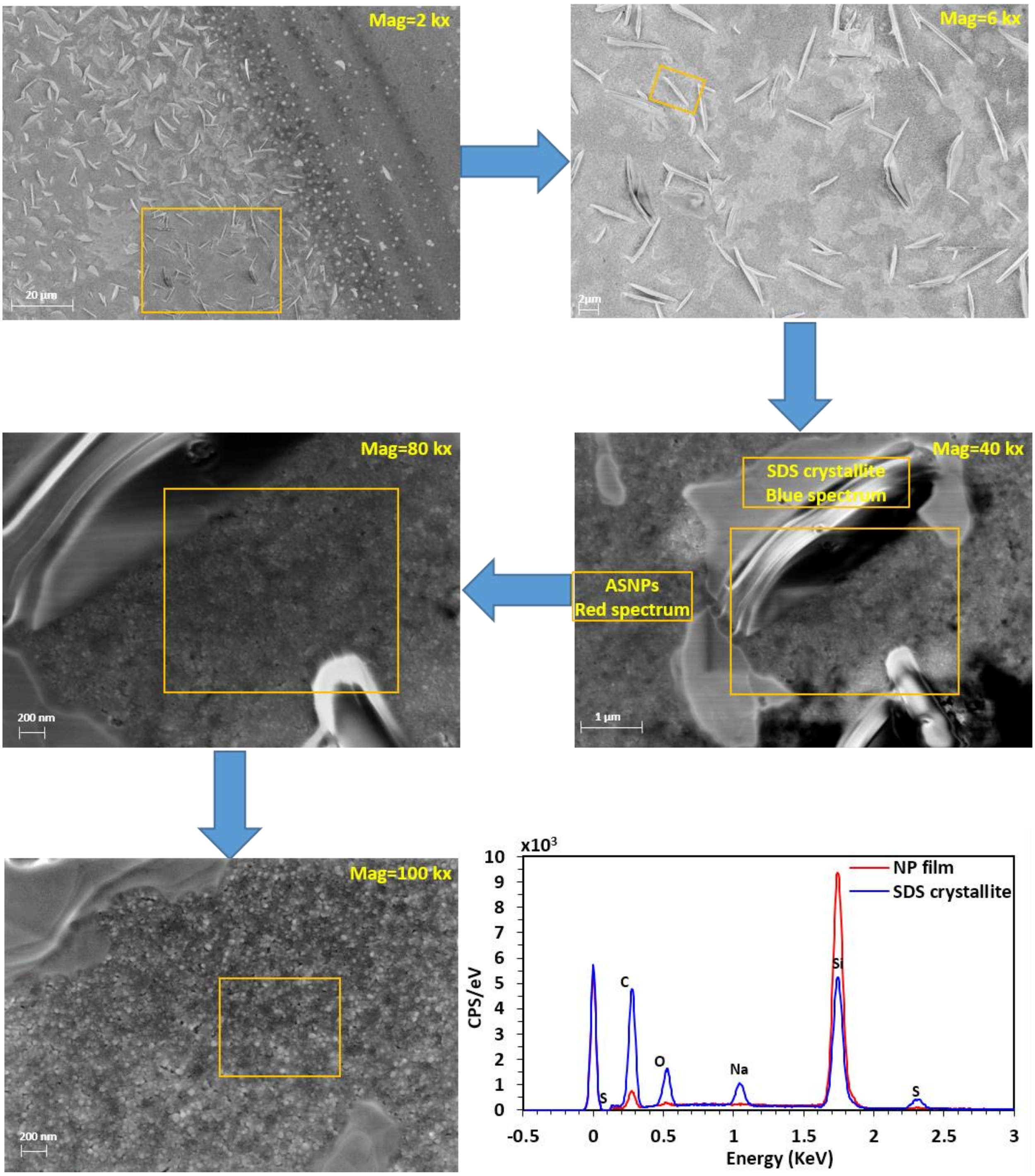

Figure S4: SEM images of P3HT:ICxA NP film with dilution factor 25 on silicon nitride for different magnifications (2, 6, 40, 80 and 100 k×). EDX spectra recorded on the SDS crystallite and nanoparticle film region.

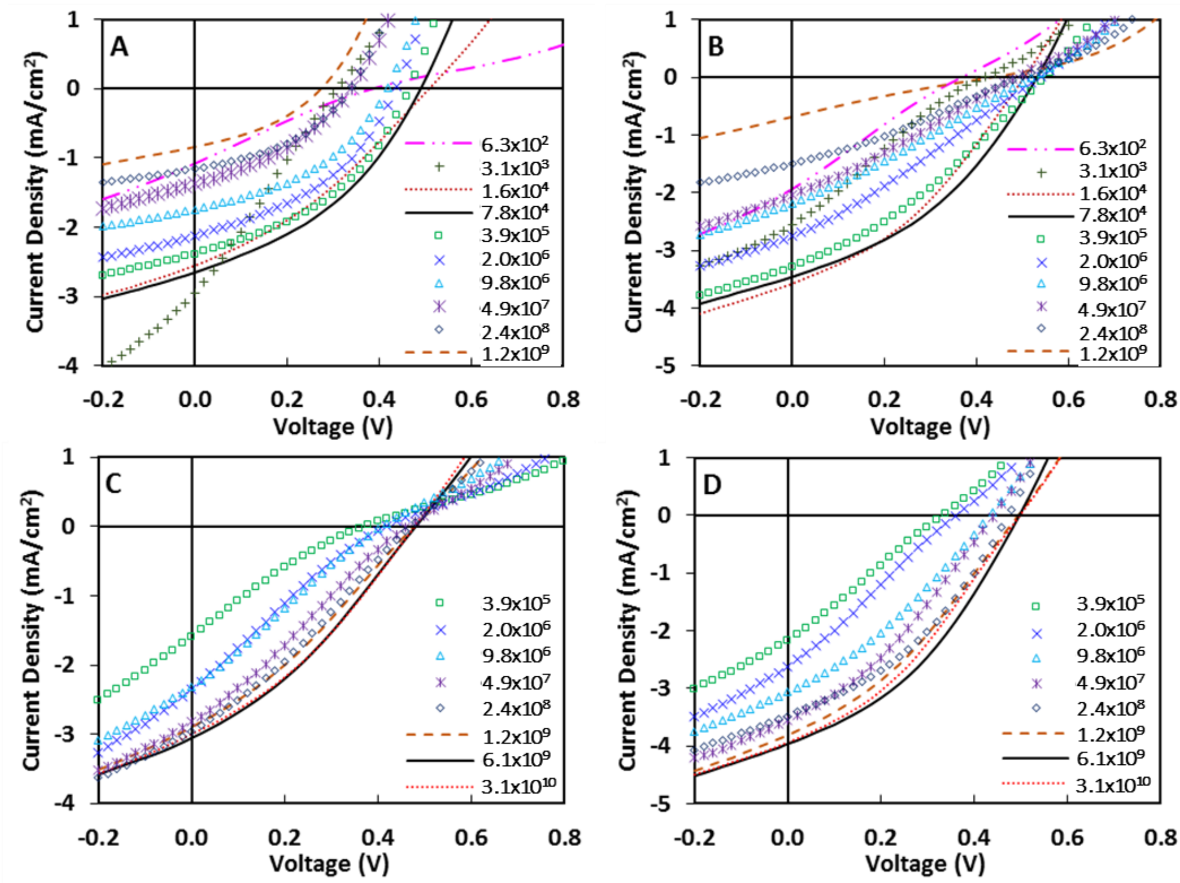

Figure S5: *J–V* characteristics curves of dried (A) and annealed (B) masked NP-OPV devices based on P3HT:ICxA NP with dilution factor of 625–1.2 × 10^9 for centrifugal process. *J–V* characteristics curves of dried (C) and annealed (D) masked NP-OPV devices based on P3HT:ICxA NP with dilution factor of 3.9 × 10^5–3.1 × 10^{10} for crossflow process.

Table S1: The performance parameters of NP-OPVs based on P3HT:ICxA NP with different dilution factor for centrifugal and crossflow ultrafiltration processes. The averages (±error) of 12 masked devices with the best masked devices (Av. ± error (Best)).

**Centrifugal ultrafiltration process**								
**OPVs (DF)**	**Dried devices**				**Annealed devices**			
	**PCE (%)**	**V_{OC} (V)**	**Fill Factor**	**J_{SC} (mA cm^{-2})**	**PCE (%)**	**V_{OC} (V)**	**Fill Factor**	**J_{SC} (mA cm^{-2})**
$6.2x10^{2}$	**0.10±0.02 (0.10)**	**0.39±0.05 (0.40)**	**0.23±0.01 (0.23)**	**1.04±0.13 (1.08)**	**0.11±0.04 (0.17)**	**0.34±0.03 (0.51)**	**0.22±0.02 (0.26)**	**1.52±0.46 (2.93)**
$3.1x10^{3}$	**0.18±0.04 (0.22)**	**0.40±0.09 (0.41)**	**0.23±0.01 (0.25)**	**1.84±0.35 (2.25)**	**0.22±0.03 (0.25)**	**0.35±0.06 (0.42)**	**0.24±0.01 (0.24)**	**2.56±0.20 (2.62)**
$1.6x10^{4}$	**0.33±0.08 (0.43)**	**0.48±0.04 (0.51)**	**0.32±0.01 (0.33)**	**2.14±0.42 (2.56)**	**0.55±0.07 (0.64)**	**0.50±0.02 (0.50)**	**0.35±0.01 (0.35)**	**3.13±0.41 (3.58)**
$7.8x10^{4}$	**0.44±0.04 (0.50)**	**0.49±0.01 (0.49)**	**0.37±0.01 (0.38)**	**2.42±0.15 (2.65)**	**0.65±0.04 (0.70)**	**0.54±0.003 (0.53)**	**0.37±0.01 (0.38)**	**3.25±0.18 (3.46)**
$3.9x10^{5}$	**0.41±0.06 (0.46)**	**0.46±0.03 (0.47)**	**0.39±0.02 (0.41)**	**2.28±0.14 (2.39)**	**0.49±0.05 (0.58)**	**0.54±0.02 (0.55)**	**0.31±0.01 (0.32)**	**2.95±0.18 (3.29)**
$2.0x10^{6}$	**0.33±0.04 (0.38)**	**0.42±0.02 (0.44)**	**0.40±0.01 (0.41)**	**1.94±0.14 (2.12)**	**0.34±0.05 (0.41)**	**0.52±0.02 (0.55)**	**0.26±0.02 (0.32)**	**2.47±0.21 (3.29)**
$9.8x10^{6}$	**0.23±0.04 (0.30)**	**0.37±0.04 (0.42)**	**0.39±0.03 (0.41)**	**1.56±0.13 (1.74)**	**0.21±0.05 (0.31)**	**0.47±0.05 (0.52)**	**0.30±0.06 (0.27)**	**1.54±0.39 (2.19)**
$4.9x10^{7}$	**0.09±0.04 (0.17)**	**0.31±0.02 (0.34)**	**0.28±0.04 (0.37)**	**0.94±0.25 (1.37)**	**0.13±0.06 (0.27)**	**0.46±0.01 (0.49)**	**0.20±0.03 (0.28)**	**1.40±0.34 (2.04)**
$2.4x10^{8}$	**0.11±0.03 (0.16)**	**0.30±0.02 (0.34)**	**0.36±0.02 (0.41)**	**0.97±0.14 (1.15)**	**0.11±0.06 (0.22)**	**0.42±0.12 (0.49)**	**0.24±0.03 (0.29)**	**1.05±0.30 (1.50)**
$1.2x10^{9}$	**0.04±0.02 (0.08)**	**0.23±0.05 (0.27)**	**0.29±0.04 (0.37)**	**0.60±0.13 (0.84)**	**0.05±0.01 (0.07)**	**0.35±0.08 (0.44)**	**0.23±0.02 (0.22)**	**0.58±0.10 (0.69)**
**Crossflow ultrafiltration process**								
**OPVs (DF)**	**Dried devices**				**Annealed devices**			
	**PCE (%)**	**V_{OC} (V)**	**Fill Factor**	**J_{SC} (mA cm^{-2})**	**PCE (%)**	**V_{OC} (V)**	**Fill Factor**	**J_{SC} (mA cm^{-2})**
$3.9x10^{5}$	**0.10±0.03 (0.14)**	**0.305±0.06 (0.351)**	**0.23±0.02 (0.22)**	**1.40 ±0.25 (-1.76)**	**0.16±0.02 (0.19)**	**0.33±0.02 (0.33)**	**0.25±0.01 (0.26)**	**1.95±0.23 (2.61)**
$2.0x10^{6}$	**0.15±0.03 (0.22)**	**0.372±0.05 (0.409)**	**0.23±0.01 (0.23)**	**1.72±0.28 (-2.35)**	**0.21±0.03 (0.25)**	**0.36±0.02 (0.36)**	**0.25±0.01 (0.26)**	**2.26±0.26 (2.62)**
$9.8x10^{6}$	**0.19±0.04 (0.24)**	**0.367±0.07 (0.418)**	**0.25±0.02 (0.23)**	**2.11±0.25 (-2.43)**	**0.28±0.08 (0.42)**	**0.37±0.08 (0.44)**	**0.27±0.02 (0.31)**	**2.78±0.21 (3.07)**
$4.9x10^{7}$	**0.27±0.04 (0.35)**	**0.427±0.02 (0.454)**	**0.27±0.01 (0.26)**	**2.36±0.23 (-2.82)**	**0.44±0.05 (0.51)**	**0.42±0.02 (0.44)**	**0.30±0.01 (0.33)**	**3.39±0.22 (3.55)**
$2.4x10^{8}$	**0.36±0.03 (0.40)**	**0.439±0.01 (0.460)**	**0.29±0.01 (0.29)**	**2.76±0.18 (-2.96)**	**0.51±0.08 (0.62)**	**0.45±0.02 (0.47)**	**0.34±0.02 (0.37)**	**3.33±0.23 (3.5)**
$1.2x10^{9}$	**0.35±0.04 (0.42)**	**0.446±0.02 (0.468)**	**0.30±0.01 (0.30)**	**2.60±0.19 (-2.90)**	**0.54±0.07 (0.63)**	**0.47±0.01 (0.50)**	**0.32±0.02 (0.33)**	**3.51±0.27 (3.81)**
$6.1x10^{9}$	**0.38±0.05 (0.47)**	**0.455±0.02 (0.472)**	**0.31±0.01 (0.32)**	**2.63±0.24 (-3.06)**	**0.67±0.04 (0.73)**	**0.50±0.01 (0.51)**	**0.36±0.01 (0.37)**	**3.64±0.22 (3.95)**
$3.1x10^{10}$	**0.39±0.05 (0.47)**	**0.459±0.03 (0.470)**	**0.32±0.01 (0.33)**	**2.57±0.23 (-3.00)**	**0.62±0.05 (0.68)**	**0.49±0.03 (0.50)**	**0.34±0.22 (0.35)**	**3.52±0.26 (3.94)**

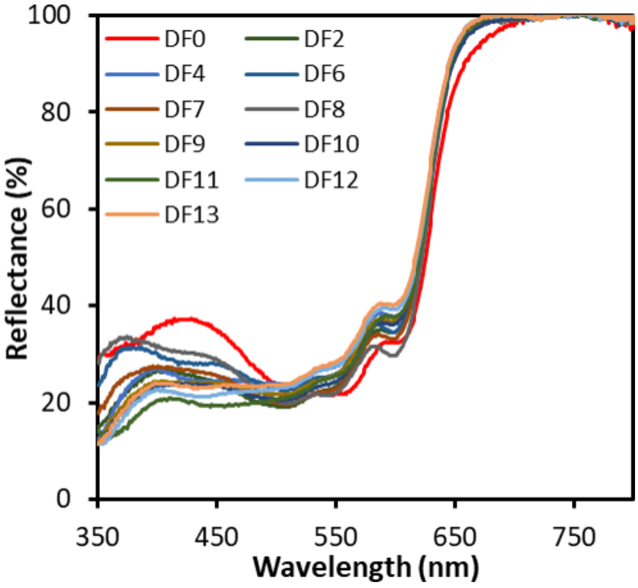

Figure S6: Reflectance measurement of the completed devices prepared from different DF inks purified by centrifuge ultrafiltration.

References

1. Retsch, M.; Zhou, Z.; Rivera, S.; Kappl, M.; Zhao, X. S.; Jonas, U.; Li, Q. *Macromol. Chem. Phys.* **2009,** 230–241. doi:10.1002/macp.200800484

2. Leunissen, M. E.; Zwanikken, J.; van Roij, R.; Chaikin, P. M.; van Blaaderen, A. *Phys. Chem. Chem. Phys.* **2007,** *9,* 6405–6414. doi:10.1039/b711300e
